# Urinary microbiome in non-muscle invasive bladder cancer: impact of sample types and sex differences

**DOI:** 10.1186/s12866-025-04367-9

**Published:** 2025-10-02

**Authors:** Chaeeun Kang, Junghoon Lee, Min-gyung Baek, Nam-Eun Kim, Hwancheol Son, Sangjun Yoo, Hana Yi

**Affiliations:** 1 Integrated Biomedical and Life Science, Graduate School, Korea University, Seoul, Korea; 2 Interdisciplinary Program in Precision Public Health, Korea University, Seoul, Korea; 3 Department of Urology, SMG-SNU Boramae Medical Center, Seoul, Korea; 4 Institute for Biomaterials, Korea University, Seoul, Korea; 5 Institute of Health and Environment, Seoul National University, Seoul, South Korea; 6 School of Biosystems and Biomedical Sciences, Korea University, Seoul, Korea

**Keywords:** Bladder cancer, Bladder microbiome, Catheterized urine, Curvibacter, Microbiome, Midstream urine, Mucosal tissue, Urinary microbiota

## Abstract

**Background:**

Previous research on urinary microbiomes in bladder cancer patients has yielded inconsistent results, highlighting the need for further investigation. This study aims to analyze microbiome dysbiosis in bladder cancer patients by comparing multiple sample types, incorporating negative controls, and assessing sex-based variations. Fifty patients who required transurethral resection of bladder tumor for treatment were selected. Three types of specimens were collected from each patient: midstream urine, catheterized urine, and normal bladder mucosal tissue. Microbiome was analyzed via 16 S rRNA gene amplificon sequencing.

**Results:**

Beta diversity analysis revealed significant differences in microbiome composition between mucosal tissue and urine samples, while no significant variation was observed between midstream and catheterized urine samples. Due to the low biomass of mucosal tissue—characterized by dominance of a few taxa and high variability across extraction kit lots—its susceptibility to contamination compromised reproducibility, leading to a focus on urine samples for further analysis. Midstream urine samples showed significant sex-related microbiome differences, whereas catheterized urine exhibited no such differences, suggesting midstream urine may not be ideal for bladder-specific microbiome studies. Catheterized urine analysis identified *Curvibacter*, particularly *Curvibacter gracilis*, as significantly more abundant in bladder cancer patients compared to controls, while overall microbiome composition remained unchanged between the groups. *Curvibacter* prevalence was not directly correlated with any single clinical marker but increased with bladder cancer severity when patients were classified into high-risk and low-risk groups based on biopsy and clinical criteria.

**Conclusions:**

This study highlights the importance of selecting appropriate sample types for bladder microbiome analysis, with catheterized urine emerging as the most reliable option. The findings suggest that *Curvibacter* may be associated with bladder cancer severity, warranting further investigation into its potential role as a biomarker. Future research should focus on validating these findings in larger cohorts and exploring the mechanistic link between microbiome alterations and bladder cancer progression.

**Supplementary Information:**

The online version contains supplementary material available at 10.1186/s12866-025-04367-9.

## Background

Bladder cancer ranks as the tenth most common cancer worldwide, predominantly affecting individuals aged 60 to 70, with men facing a higher risk than women. Smoking is a well-established major risk factor. Despite a relatively favorable five-year survival rate of 70–90%, the high recurrence rate in high-risk patients significantly affects their quality of life [1].

A wide range of biomarkers, including proteins, nucleic acids, and metabolic signatures, has been investigated for bladder cancer diagnosis and surveillance using blood, urine, and tissue samples [2]. Although several approved assays are available, their clinical utility is limited by inconsistent sensitivity and specificity. To address this, multi-biomarker panels that combine molecular and cellular markers are required for early detection and prognosis [3]. The microbiome could be one such candidate.

Recent studies have identified the existence of a normal urinary bladder microbiome, underscoring the need to explore its relationship with various diseases, including bladder cancer [4–6]. Consequently, the urinary microbiome has emerged as a novel biomarker candidate, as its composition and functional profiles are increasingly recognized to influence bladder cancer development, progression, and therapeutic responses [7, 8].

In other solid tumors, the gut microbiome has been recognized for its role in modulating immune responses and influencing the efficacy of immune checkpoint inhibitor therapy [9]. Specific bacterial species have been linked to treatment outcomes, and ongoing research is investigating their potential as both predictive biomarkers and therapeutic targets. These insights highlight the importance of characterizing the bladder microbiome, not only to elucidate its role in carcinogenesis but also to assess its potential as a diagnostic and prognostic tool.

Because urine contains a low microbial biomass than other human samples, rigorous sampling, storage, and preprocessing procedures are essential to minimize contamination and accurately capture the true bladder microbiome. Furthermore, the choice of sample type—midstream urine, catheterized urine, or bladder mucosal tissue—can substantially influence the observed microbiome composition. Previous studies have begun to examine differences between urine and bladder tissue, as well as between bladder cancer patients and non-cancer controls. However, findings to date have been inconsistent, and few investigations have simultaneously assessed multiple sample types while also accounting for potential sex-related differences.

In this study, we analyzed the bacteriome of bladder cancer patients and a non-bladder cancer control group to identify differences in microbiome composition. To determine the optimal sample type for bladder cancer microbiome analysis, we collected and compared midstream urine, catheterized urine, and bladder mucosal tissue samples. Multiple negative controls were included to distinguish true microbiomes from contaminant communities. Furthermore, sex-based subgroup analysis was conducted for midstream and catheterized urine samples to evaluate sex-related differences in the bladder microbiome.

## Materials and methods

### Study subjects

This study was approved by the Institutional Review Board of Seoul National University Boramae Medical Center (IRB No. 10-2022-9). Patients presenting with a bladder mass or with suspected bladder cancer based on cystoscopy and/or imaging studies were initially screened. A total of 50 patients who required transurethral resection of bladder tumor (TURBT) were ultimately enrolled. Among them, 41 were newly diagnosed cases, while 9 had a prior history of TURBT. Written informed consent was obtained from all participants after a detailed explanation of the study procedures.

### Sample collection

Three types of specimens were collected from each patient: midstream urine, catheterized urine, and normal bladder mucosal tissue. Midstream urine was collected a day before surgery using the clean-catch method. The other specimens were obtained intraoperatively under general anesthesia during TURB. Prior to cystoscope insertion, a clean intermittent catheter was used to collect catheterized urine. Bladder mucosal biopsies were taken from the posterior wall, at least 3 cm away from the tumor site, using cystoscopic biopsy forceps. Baseline patient characteristics, including recent history of cystitis, antibiotic use, and probiotic intake, were recorded. Results of urine and blood tests, along with pathological findings, were also collected.

Urine specimens (midstream and catheterized) were collected in 50 ml Falcon tubes and preserved with 5 ml of AssayAssure™ RNA-stabilizing reagent (Sierra Molecular Inc., Incline Village, NV, USA, Cat No. 14440) at a 10% ratio. All specimens were refrigerated prior to analysis, and DNA extraction was performed within three weeks of collection. Tissue specimens were immediately frozen after collection and stored at −80 °C to prevent microbiome alterations.

### Sample processing

To inhibit microbial growth, samples were processed at 4 °C. Urine samples were centrifuged at 14 g for 10 min, and the supernatant was transferred. Remaining human cells were removed using a 5 μm pore-sized syringe filter (Sartorius Stedim Biotech, Goettingen, Germany, Cat No. S7594). Bacterial cells were concentrated using a Vivaspin^®^ 20 ultrafiltration device (Sartorius Stedim Biotech, Goettingen, Germany, Cat No. VS2021) with a 30,000 kDa pore-sized polyethersulfone membrane. Ultrafiltration was conducted at 5,000 g until a final volume of less than 500 µL remained. The bacterial cells were de-salted by adding 5 ml of sterile 1X Phosphate Buffered Saline (PBS, Biosesang, Korea, Cat No. PR2004-100-72) twice. The de-salted bacterial cells were resuspended in 978 µL of sodium phosphate buffer and 122 µL of lysis buffer (MP Biomedicals, USA, Cat No. 6560200).

### Experimental negative controls

Given the low biomass of the bladder microbiome, contamination from external microorganisms introduced via surgical instruments, experimental kits, and reagents is a significant concern. To address this, all surgical instruments, sample collection tools, laboratory consumables, and reagents used were designated as negative controls. Reusable surgical instruments were swabbed with sterile swabs soaked in lysis buffer and immersed in 1.5 ml tubes containing lysis buffer. Single-use instruments, such as catheters, were cut into pieces with sterile scissors and immersed in lysis buffer. Nucleic acids from these negative controls were extracted and sequenced using the same protocols applied to clinical samples. A total of 30 negative control samples were analyzed.

### DNA extraction and 16 S rRNA gene sequencing

Metagenomic DNA was extracted using the FastDNA™ SPIN Kit for Soil (MP Biomedicals, USA, Cat No. 116560-200) following the manufacturer’s protocol. PCR amplification of the V3-V4 hypervariable region of the bacterial 16 S rRNA gene was performed using primers 341 F (5’-TCGTCGGCAGCGTCAGATGTGTATAAGAGACAGCCTACGGGNGGCWGCAG-3’) and 805R (5’-GTCTCGTGGGCTCGGAGATGTGTATAAGAGACAGGACTACHVGGGTATCTAATCC-3’). The reaction mixture consisted of 3 µL extracted DNA, 1 µL of each 20 µM primer, and 25 µL of 2X KAPA HiFi HotStart ReadyMix (KAPA Biosystems, Resnova, Rome, Italy). PCR conditions included an initial denaturation at 95 °C for 3 min, followed by 25 cycles of denaturation at 95 °C for 30 s, annealing at 65 °C for 30 s, and extension at 72 °C for 30 s, with a final extension at 72 °C for 5 min. PCR products were purified using Agencourt AMPure XP Beads (Beckman Coulter, Inc., CA, USA, Cat No. A63881). Indexed PCR was performed using the Nextera Index Kit (Illumina, CA, USA), followed by a second round of purification with AMPure XP Beads. The final library was quantified using a DeNovix fluorometer (DeNovix Inc., USA) with the DeNovix dsDNA Broad Range Assay, and library size distribution was assessed using the 4150 TapeStation System with D1000 ScreenTape (Agilent Technologies, Santa Clara, USA). Sequencing was conducted on the Illumina MiSeq v3 platform (Illumina, CA, USA).

### Data processing and statistical analysis

Sequencing reads were processed using the QIIME2 pipeline (version 2021.4) with taxonomy assignments based on the EzTaxon-e database. Demultiplexing was conducted to separate individual samples, and DADA2 was applied to filter low-quality and duplicate sequences. Alpha-diversity was assessed using Chao1, ACE, Shannon, and Simpson indices. Beta-diversity was calculated using the Bray-Curtis distance metric, with log CPM values adjusted for batch effects and normalization. Principal coordinate analysis (PCoA) was performed using the R package “vegan,” and statistical significance was evaluated with permutational multivariate analysis of variance (PERMANOVA). The Benjamini-Hochberg correction was applied to adjust for multiple comparisons. Taxonomic differences were analyzed using ANCOM, linear regression, and logistic regression. Amplicon sequence variants (ASVs) were identified using QIIME2 and manually verified with the EzBioCloud database.

### Batch effect correction and normalization

Due to a time gap in sample collection between two sets of participants (*n* = 43 and *n* = 7), different lot numbers of nucleic acid extraction kits were used. To account for potential batch-related variation—particularly critical in low-biomass samples—batch effect correction and normalization were applied. Principal component analysis (PCA) was conducted using the “pcaMethods” R package to identify technical biases. The Combat method from the “sva” R package was applied to correct batch effects, with log-transformed CPM values used for normalization before processing.

## Results

### Clinical and demographic characteristics of study participants

Baseline characteristics of the study population are summarized in Table 1. The cohort included 50 participants with bladder mass on cystoscopy and/or imaging studies who underwent transurethral surgery. After surgery, these patients were divided into 2 groups based on pathologic diagnosis (35 patients with bladder cancer [bladder cancer] vs. 15 patients without cancer [non-cancer]). No significant differences were observed in age or body mass index (BMI) between the two groups. White blood cell (WBC) counts were moderately lower in the bladder cancer group compared to controls, approaching statistical significance (*p*-value = 0.0539). Other clinical parameters did not differ significantly between groups. Detailed clinical data relevant to bladder cancer were collected exclusively for the patient group.

**Table 1:** Clinical and demographic characteristics of study participants

Characteristics	Bladder Cancer (*n* = 35)	Non-Cancer (*n* = 15)	*p*-value
Sex, n (%)			
Female	6 (17.1)	2 (13.3)	
Male	29 (82.9)	13 (86.7)	
Age, mean ± SD (years)	67.6 ± 9.9	67.2 ± 12.1	0.8237
BMI, mean ± SD (kg/m^2^)	25.3 ± 2.8	25.3 ± 2.5	0.8159
Diabetes, n (%)	11 (31.4)	6 (40.0)	Ns
Hypertension, n (%)	18 (51.4)	8 (53.3)	Ns
Recent antibiotics usage, n (%)	1 (2.9)	0 (0.0)	Ns
Recent cystitis, n (%)	3 (8.6)	1 (6.7)	Ns
Probiotics usage, n (%)	6 (17.1)	2 (13.3)	Ns
WBC, mean ± SD (/µL)	6190 ± 1549	6995 ± 917	0.0539
ESR, mean ± SD (mm/hr)	21.1 ± 15.6	18.6 ± 11.6	0.8183
Pathologic Stage, n (%)			
CIS only	4 (11.4)		
Ta	26 (74.3)		
T1	5 (14.3)		
Tumor Grade, n (%)			
Low	18 (51.4)		
High	17 (48.6)		
Tumor Size, n (%)			
< 3 cm	28 (80.0)		
≥ 3 cm	7 (20.0)		
Tumor Multiplicity, n (%)	21 (60.0)		
AUA Risk Classification, n (%)			
Low	11 (31.4)		
Intermediate	9 (25.7)		
High	15 (42.9)		

Summary of patient demographics and clinical variables in the bladder cancer (*n* = 35) and non-cancer (*n* = 15) groups, including age, sex, comorbidities, laboratory parameters, and tumor characteristics. Values are presented as number (percentage) or mean ± standard deviation. *P*-values were calculated using Student’s t-test or chi-square test, as appropriate

*NS* Not significant, *WBC* White blood cell count, *ESR* Erythrocyte sedimentation rate, *CIS* Carcinoma in situ, *AUA* American Urological Association

### Sequencing statistics

In this study, three types of samples were collected from each participant for analysis. The mean raw reads per sample were 158,307, with averages of 197,379, 182,561, and 95,765 for midstream urine, catheterized urine, and mucosal tissue, respectively. This indicates that sequencing reads were higher in urine samples compared to mucosal tissue. The average read count of experimental negative controls was 60,918, which was lower than that of the clinical samples.

### Kitomes detected in experimental negative controls

Sequencing analysis of experimental negative controls identified diverse bacterial composition. *Escherichia* and *Stenotrophomonas* accounted for 60% of the identified sequences, and other bacteria including *Rhodococcus*, *Streptococcus*, and *Pseudomonas* accounted for the remaining portion. Samples derived from latex materials, such as rubber packing and latex catheters, were primarily composed of *Stenotrophomonas*, while surgical instruments showed a predominant presence of *Escherichia*. Non-sterile items, such as biopsy containers and urine sample collection containers, exhibited more diverse microbial communities. Minimal contamination was observed in PCR reagents. Given these findings, careful interpretation of bladder microbiome study results is crucial, as several bacteria identified in negative controls may appear as contaminants or “kitome.” A detailed list of the negative control samples is provided in Supplementary Table 1, and the bacterial composition of each sample is illustrated in Supplementary Fig. 1.

### Microbiome differences by specimen type

**Fig. 1:**
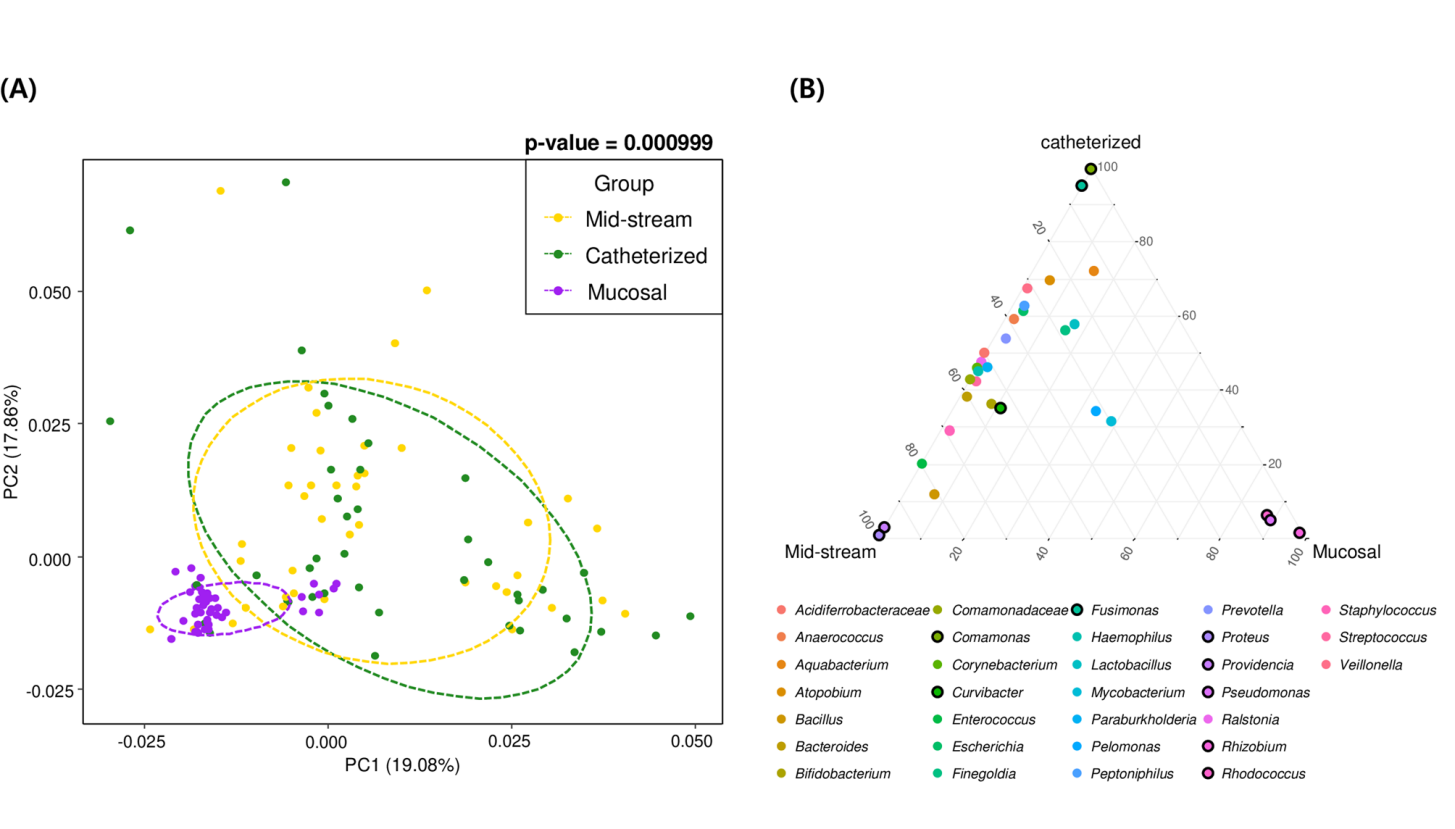
Microbiome composition by specimen type in male patients. **A** PCoA plot of beta diversity based on Bray-Curtis distances, demonstrating significant differences in microbiome composition between urine and mucosal tissue samples in male patients. Statistical significance was assessed using PERMANOVA, with ellipses representing the 65% confidence interval. **B** Ternary plot of average genus-level compositions across three sample type groups. Genera are positioned based on relative abundance in each sample type, highlighting distinct enrichment patterns by specimen

To minimize sex-related bias, microbiome characteristics were first analyzed separately for male patients (*n* = 42). Beta diversity analysis revealed significant differences among the three sample types (midstream urine, catheterized urine, and mucosal tissue) (*p*-value = 0.000999 ***) (Fig. 1A). However, when comparing the two urine samples, no statistically significant difference was found (*p*-value = 0.1449). This suggests that mucosal tissue samples exhibit a distinct microbiome composition compared to urine samples. Distinct bacterial taxa were found to be specifically associated with each sample type. In particular, the mucosal tissue samples were dominated by *Rhodococcus*, *Rhizobium*, and *Pseudomonas*. The two urine sample types also exhibited biased distributions of certain taxa: *Proteus* and *Providencia* were characteristic of midstream urine, while *Comamonas* and *Fusimonas* were more prevalent in catheterized urine (Fig. 1B). The dominant genera in tissue samples are detected in our experimental negative controls and also frequently reported as contaminants in low-biomass microbiome studies, suggesting that mucosal tissue may contain minimal native microbiota and be unsuitable for bladder microbiome analysis.

A similar trend was observed in female participants (*n* = 8), where tissue samples showed a significant difference from urine samples (*p*-value = 0.01598 *), with the dominant genera remaining consistent (Supplementary Fig. 2). No significant difference was observed between midstream and catheterized urine samples (*p*-value = 0.8921), aligning with findings in male participants. When data from both sexs were analyzed together, the tissue samples remained distinct from the urine samples, with no significant differences between the two urine types (Supplementary Fig. 3). Therefore, subsequent analyses focused on urine samples.

### Effect of sex on the urine Microbiome

**Fig. 2:**
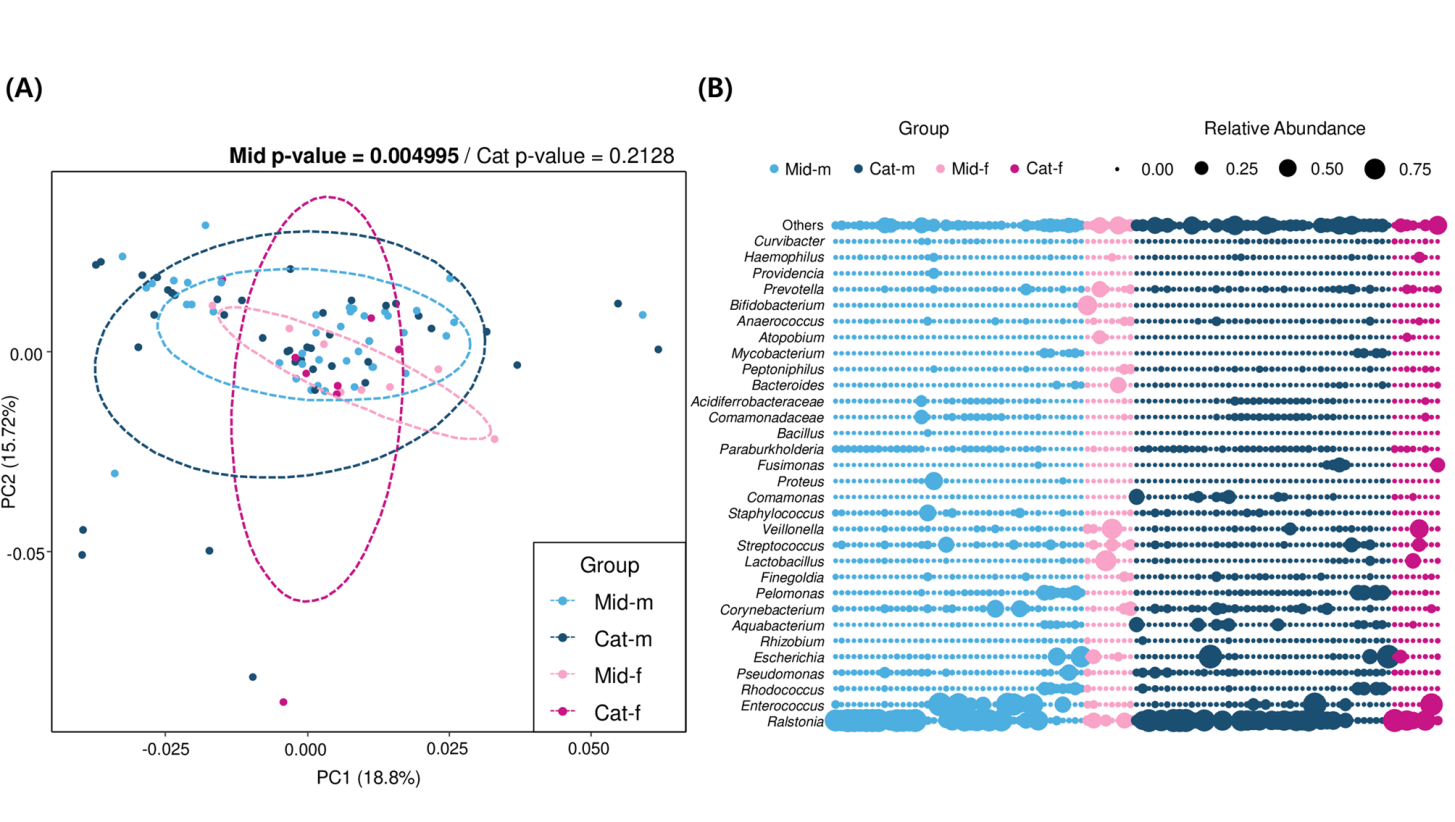
Sex-related differences in midstream and catheterized urine microbiome. **A** PCoA plot of beta diversity based on Bray-Curtis distances, illustrating greater sex-related variation in midstream urine compared to catheterized urine. Statistical significance was assessed using PERMANOVA, with ellipses representing the 65% confidence interval. **B** Bubble plot of genus-level relative abundances across samples, highlighting sex- and method-specific microbiome patterns among the four urine groups

Due to anatomical differences in the urogenital tract, sample collection methods differ between men and women. Accordingly, midstream and catheterized urine samples were analyzed separately by sex. Beta diversity analysis showed no significant differences between males and females in catheterized urine samples (*p*-value = 0.2128). However, significant differences were observed in midstream urine samples (*p*-value = 0.004995 ***) (Fig. 2A). In the female group, *Lactobacillus* was more abundant across urine samples compared to the male group. Specifically, female midstream urine samples exhibited decreased levels of *Ralstonia* (*p*-value = 0.0186) and increased levels of *Bacteroides* (*p*-value = 0.0949) and *Bifidobacterium* (*p*-value = 0.0781) relative to other groups (Fig. 2B). These findings indicate that midstream urine is significantly influenced by anatomical differences and may not be an ideal sample type for bladder-specific microbiome studies, such as those focusing on urothelial carcinoma.

### Bacteria associated with urothelial carcinoma

**Fig. 3:**
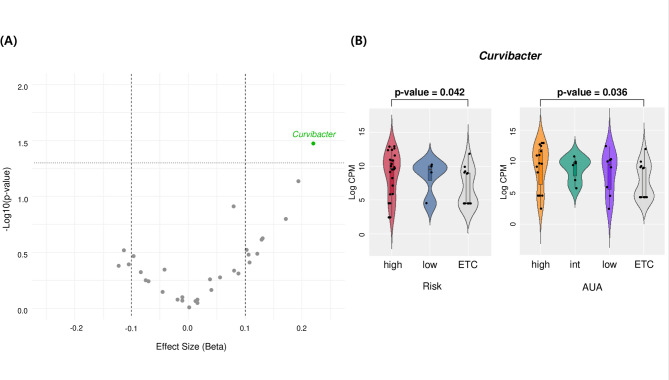
*Curvibacter* is associated with urothelial carcinoma and its clinical severity. **A** Volcano plot highlighting *Curvibacter* as the only genus significantly associated with urothelial carcinoma (UC) compared to non-cancer controls with cancer-like symptoms (ETC) (*p* < 0.05 and |effect size| >0.1). **B** Violin plots showing *Curvibacter* abundance across UC risk groups (left) and AUA severity categories (right). Significant differences were observed in both comparisons, with *p*-values adjusted using the Benjamini-Hochberg method. Boxplot whiskers indicate the data range, with boxes representing the interquartile range and the line denoting the median

After assessing the effects of sample type and sex, catheterized urine was identified as the most suitable sample type for bladder microbiome analysis. To minimize sex-related bias, differences between bladder cancer patients (*n* = 29) and non-bladder cancer controls (*n* = 13) were initially analyzed using male catheterized urine samples. Beta diversity analysis revealed no significant difference between the two groups (*p*-value = 0.1319), suggesting that bladder cancer does not substantially alter the overall microbiome composition. However, independent analyses using ANCOM-BC and linear regression identified *Curvibacter* as the only bacterial genus showing a significant difference between the groups. *Curvibacter* was more abundant in bladder cancer patients compared to controls, with statistical significance (ANCOM-BC *p*-value = 0.035) (Fig. 3A). When the analysis was restricted to the female catheterized urine subgroup, *Curvibacter* also appeared more prevalent in bladder cancer patients, although the difference did not reach statistical significance (ANCOM-BC *p*-value = 0.516). At species-level, *Curvibacter gracilis* was the dominant species composing 86% of the *Curvibacter* sequences in bladder cancer samples and 60% in non-bladder cancer samples.

### Association between Curvibacter and clinical features

To further investigate the relationship between *Curvibacter* prevalence and clinical indicators, ANCOM-BC analysis was performed on clinical metrics listed in Table 1. No individual clinical marker showed a direct correlation with *Curvibacter* levels. Consequently, patients with bladder cancer were reclassified into high-risk and low-risk bladder cancer groups based on the pathologic and clinical features. Additional classifications were made according to the AUA criteria for recurrence and progression of non-muscle invasive bladder cancer. Ordinal logistic regression analysis indicated that *Curvibacter* prevalence increased with bladder cancer severity in both the Risk and AUA clinical groupings (*p*-value < 0.05) (Fig. 3B). These findings were further validated using multinomial logistic regression and the Kruskal-Wallis test. Multinomial logistic regression revealed statistically significant differences in *Curvibacter* prevalence across both the Risk and AUA groups (*p* < 0.05). Although the Kruskal-Wallis test did not yield statistically significant results, mean *Curvibacter* abundance showed a consistent increasing trend with disease severity.

## Discussion

*Curvibacter* is a genus of Gram-negative bacteria that includes species such as *C. delicates*,* C. fontanus*,* C. gracilis*, and *C. lanceolatus*. These bacteria are primarily found in aquatic environments, such as well water, but they have also been detected in animal and human samples. Several studies have examined the presence of *Curvibacter* in human microbiomes. Gedgaudas et al. [10] reported a significant reduction of *Curvibacter* in the plasma microbiome of cirrhosis patients, while Jiang et al. [11] found lower abundance in the esophageal tissue microbiome of esophageal squamous cell carcinoma patients compared to healthy individuals. The observed increase of *Curvibacter* in urine samples from bladder cancer patients is noteworthy, suggesting a possible association with bladder cancer. Ning et al. [12] found a significant reduction of *Curvibacter* in the urine microbiome of gout patients compared to healthy controls, indicating that its distribution in urine may vary with different disease conditions.

Tong et al. [13] reported that bacteria can degrade uric acid via urate oxidase (uricase) or xanthine dehydrogenase inhibitory activity. Certain bacteria, such as *Lactobacillus*, inhibit uric acid biosynthesis, whereas *Escherichia-Shigella* contributes to uric acid production by secreting xanthine deaminase. Our EggNOG analysis of the genes of *Curvibacter gracilis*, which was the predominant *Curvibacter* species in our study, confirmed the presence of xanthine dehydrogenase (XDH), an enzyme involved in purine metabolism and uric acid production. Chen et al. [14] reported that elevated uric acid levels are an independent risk factor for bladder cancer-specific survival, suggesting that *Curvibacter*’s presence in the bladder may contribute to increased uric acid concentrations and potentially influence bladder cancer progression.

With the recognition that the bladder is not a sterile environment, research on the bladder and urinary microbiome has expanded considerably. Early studies primarily relied on voided midstream urine samples, but more recent work has shifted towards catheterized urine to minimize external contamination. Oresta et al. [15] compared midstream urine, catheterized urine, and bladder washout urine collected from bladder cancer patients and control groups, reporting that midstream urine was enriched in skin-associated bacteria such as *Streptococcus* and *Corynebacterium*, whereas bladder washout urine contained a higher abundance of gut-associated taxa including *Faecalibaculum* and *Erysipelatoclostridium*. Consistent with these findings, we observed sex-specific differences in midstream urine but not in catheterized urine, supporting the use of catheterized samples as a more reliable source for bladder microbiome analysis.

Research has also compared the microbiome of bladder tissue and urine. Mansour et al. [16] analyzed samples from bladder cancer patients undergoing transurethral resection and reported age- and sex-related differences between urine and bladder tissue, with *Akkermansia* and *Klebsiella* being more prevalent in tissue samples. Similarly, Liu et al. [17] observed that male patients had higher relative abundances of *Lactobacillus* in normal tissues, whereas cancerous tissues contained more pathogenic genera such as *Acinetobacter* and *Escherichia-Shigella*. Additional studies comparing tumor and non-tumor tissues have shown that tumor tissues are enriched for *Burkholderia* in both sexes [18]while non-tumor tissues exhibit higher alpha diversity and greater abundance of Actinomycetota [19]. Consistent with these findings, our dataset revealed that *Rhodococcus*, a genus within Actinomycetota, accounted for an average of 35% of tissue samples across both sexes, indicating a striking dominance in line with previous reports. Taken together, these results suggest that bladder mucosal tissues are often dominated by a limited number of taxa—such as *Rhodococcus*, *Pseudomonas*, and *Rhizobium*—many of which have frequently been reported as contaminants in low-biomass studies. This reinforces concerns that tissue-based analyses are highly susceptible to contamination, suggesting that bladder mucosa may harbor only a minimal indigenous microbiota.

Comparative studies of urinary microbiomes between bladder cancer patients and non-cancer individuals, including those with benign urological conditions, have yielded inconsistent results. For instance, Bucevic Popovic et al. [20] reported that *Veillonella* and *Corynebacterium* were more abundant in the controls, whereas Oresta et al. [15] found higher levels in bladder cancer patients. Similarly, Hrbacek et al. [21] observed that *Veillonella* was more prevalent in bladder cancer, while *Corynebacterium* was more common in controls. Such inconsistencies underscore the complexity of the urinary microbiome’s role in bladder cancer. To our knowledge, however, no prior study has simultaneously compared midstream urine, catheterized urine, and bladder mucosal tissue samples while explicitly addressing sex-based microbiome differences. Our findings suggest that catheterized urine provides a more reliable representation of bladder-associated microbiota across sexes, while also highlighting the methodological challenges of interpreting results derived from tissue samples and midstream urine.

In this study, three sample types—midstream urine, catheterized urine, and bladder mucosal tissue—were collected and compared within individuals. No significant differences in beta diversity were observed between midstream and catheterized urine samples, either when all participants were analyzed together or when stratified by sex (male: *p* = 0.1469; female: *p* = 0.8911). However, a significant sex-based variation was detected in midstream urine samples, whereas catheterized urine samples showed no such difference. These findings suggest that midstream urine is susceptible to sex-related contamination, likely from genital and skin flora, while catheterized urine provides a more consistent representation of the bladder microbiome across sexes.

The microbial composition of bladder mucosal tissue samples differed markedly from that of urine samples, with *Rhodococcus*, *Pseudomonas*, and *Rhizobium* being the predominant genera. According to Salter et al. [22] these microorganisms have been commonly reported as contaminants in low-biomass microbiome studies, suggesting that the bladder mucosa likely contains minimal indigenous microbiota. In the present study, a batch effect arising from differences in the lot numbers of nucleic acid extraction kits resulted in distinct microbial community profiles between the 43 and 7 tissue samples. Notably, the 7 samples clustered closely with the kitome control in the PCoA plot based on beta diversity (Fig. S4), indicating a strong influence from kit-derived contaminants. These findings highlight the susceptibility of tissue samples to technical variability depending on the extraction kit used, thereby raising concerns about reproducibility. Therefore, special caution must be taken to minimize contamination when using mucosal tissue as a sample type in microbiome research.

Unlike previous studies, our investigation employed rigorous storage and processing techniques to minimize microbial distortion. Urine samples were mixed with preservative and refrigerated immediately, avoiding storage at −80 °C to prevent microbial damage due to crystallization. The use of a spin concentrator improved the recovery of microbial cells, addressing the limitations of low-biomass samples. Such methodological considerations enhance the reliability of our findings and may serve as a practical framework for future studies of the bladder microbiome.

## Conclusions

This study highlights the presence of *Curvibacter gracilis* in the bladder microbiome of bladder cancer patients and its potential role in uric acid metabolism. Comparative analysis of sample types showed that midstream urine may be affected by sex-related contamination, while mucosal tissue is susceptible to kit-derived artifacts. In contrast, catheterized urine samples provided more consistent and reliable microbial profiles. These findings support the use of catheterized urine as a preferred sample type for bladder microbiome studies, while emphasizing the need to account for sample-specific biases in future research.

However, this study has several limitations. First, the control group comprised patients with benign conditions rather than healthy individuals, which may limit the generalizability of the findings. Second, the overall sample size was relatively small, particularly the limited number of female participants (*n* = 8), which substantially reduced the statistical power of sex-based analyses. Third, as we analyzed normal bladder mucosa instead of tumor tissue, the results offer only limited insight into the tumor-specific microbiome.

## Supplementary Information

Supplementary Material 1.

Supplementary Material 2.

Supplementary Material 3.

Supplementary Material 4.

Supplementary Material 5.

## Data Availability

The raw sequence data of the samples were deposited in the Sequence Read Archive database (BioProject accession number PRJNA1248049).
