# Supplementary material for: Urinary microbiome in non-muscle invasive bladder cancer: impact of sample types and sex differences
Source: BMC Microbiol. 2025 Oct 2;25:623. doi: 10.1186/s12866-025-04367-9 (PMC12492864; doi:10.1186/s12866-025-04367-9)
Supplement: Supplementary file 1 — Supplementary Material 1. [file 12866_2025_4367_MOESM1_ESM.docx]

**Supplementary Table 1. List of the negative control**

| **Sample** | **Material** |
| --- | --- |
| N001 | Newly opened lidocaine jelly |
| N002 | Used lidocaine jelly |
| N003 | Inside of the jaw (biopsy forcep) |
| N004 | Outside of the jaw (biopsy forcep) |
| N005 | Shaft (biopsy forcep) |
| N006 | Biopsy forcep (directly immersed in MT buffer) |
| N007 | Distal part of cystoscope |
| N008 | Cystoscope (directly immersed in MT buffer) |
| N009 | Irrigation inlet |
| N010 | Irrigation outlet |
| N011 | Distal part of external sheath |
| N012 | Proximal part of external sheath |
| N013 | Distal part of bridge |
| N014 | Proximal part of bridge 1 |
| N015 | Proximal part of bridge 2 |
| N016 | Rubber sealing cap |
| N017 | Obturator |
| N018 | Swab + Kit |
| N019 | Kit (kitome) |
| N020 | Latex glove |
| N021 | IV administration set |
| N022 | Latex catheter |
| N023 | Foley catheter |
| N025 | Lid of specimen container |
| N027 | Specimen container 1 |
| N028 | Specimen container 2 |
| N031 | Normal saline 50ml (Vivaspin) |
| N032 | Assayassure 5ml (centrifuged pellet) |
| N033 | 1^st^ PCR negative control |
| N034 | 2^nd^ PCR negative control |
