# Supplementary figures and images for: Urinary microbiome in non-muscle invasive bladder cancer: impact of sample types and sex differences
Source: BMC Microbiol. 2025 Oct 2;25:623. doi: 10.1186/s12866-025-04367-9 (PMC12492864; doi:10.1186/s12866-025-04367-9)

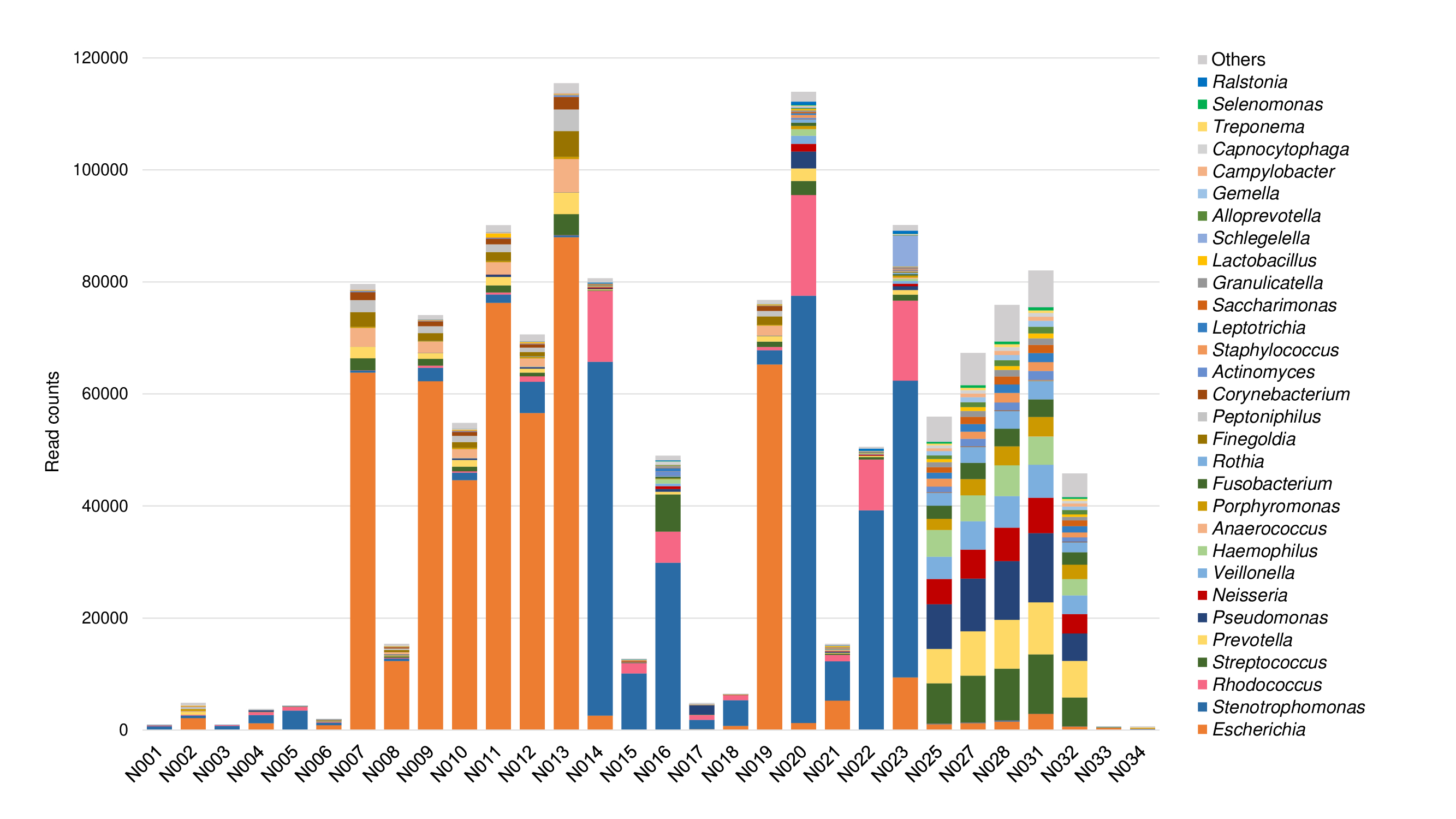

Supplement: Supplementary file 2 — Supplementary Material 2. [file 12866_2025_4367_MOESM2_ESM.jpg]

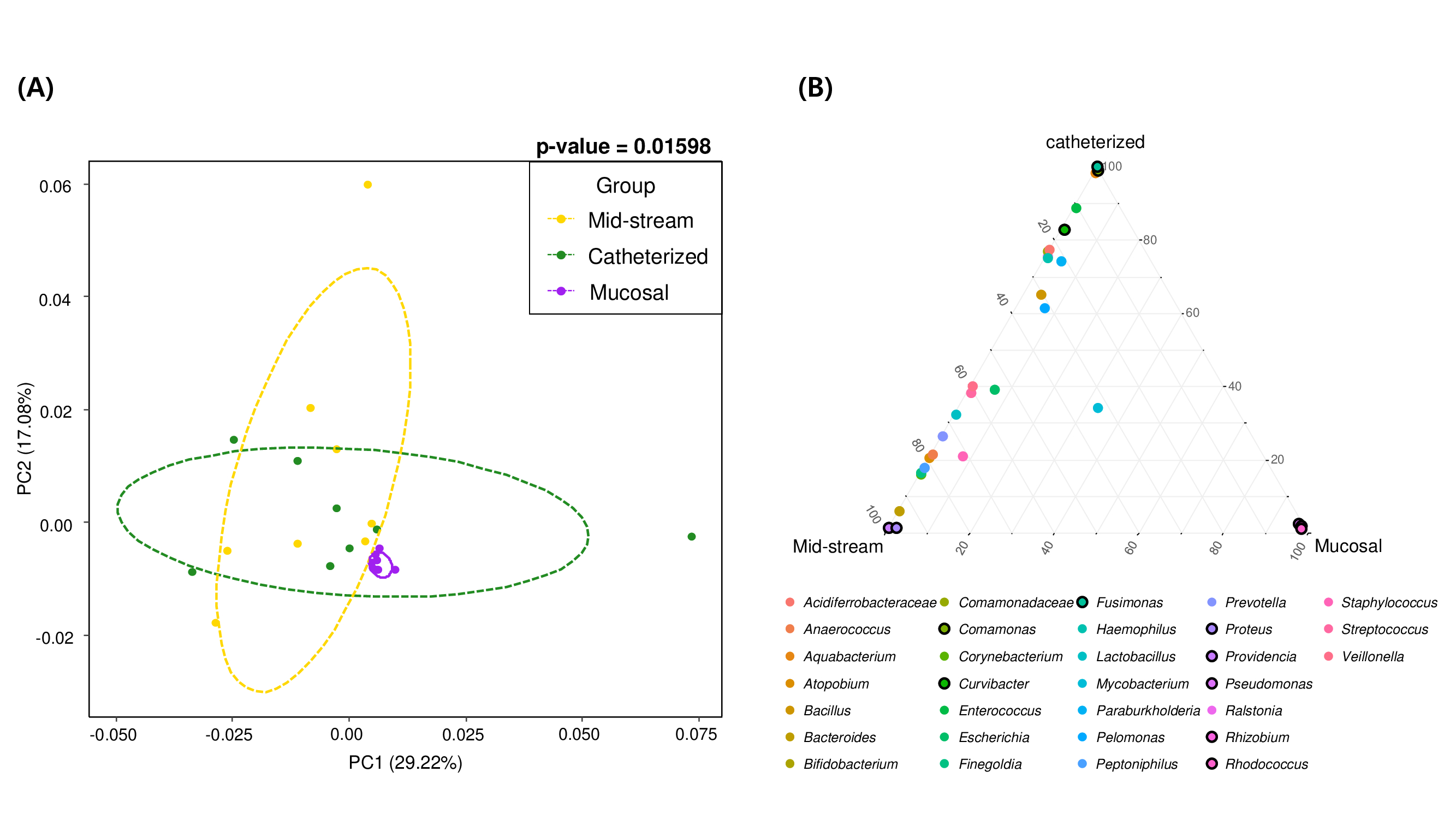

Supplement: Supplementary file 3 — Supplementary Material 3. [file 12866_2025_4367_MOESM3_ESM.jpg]

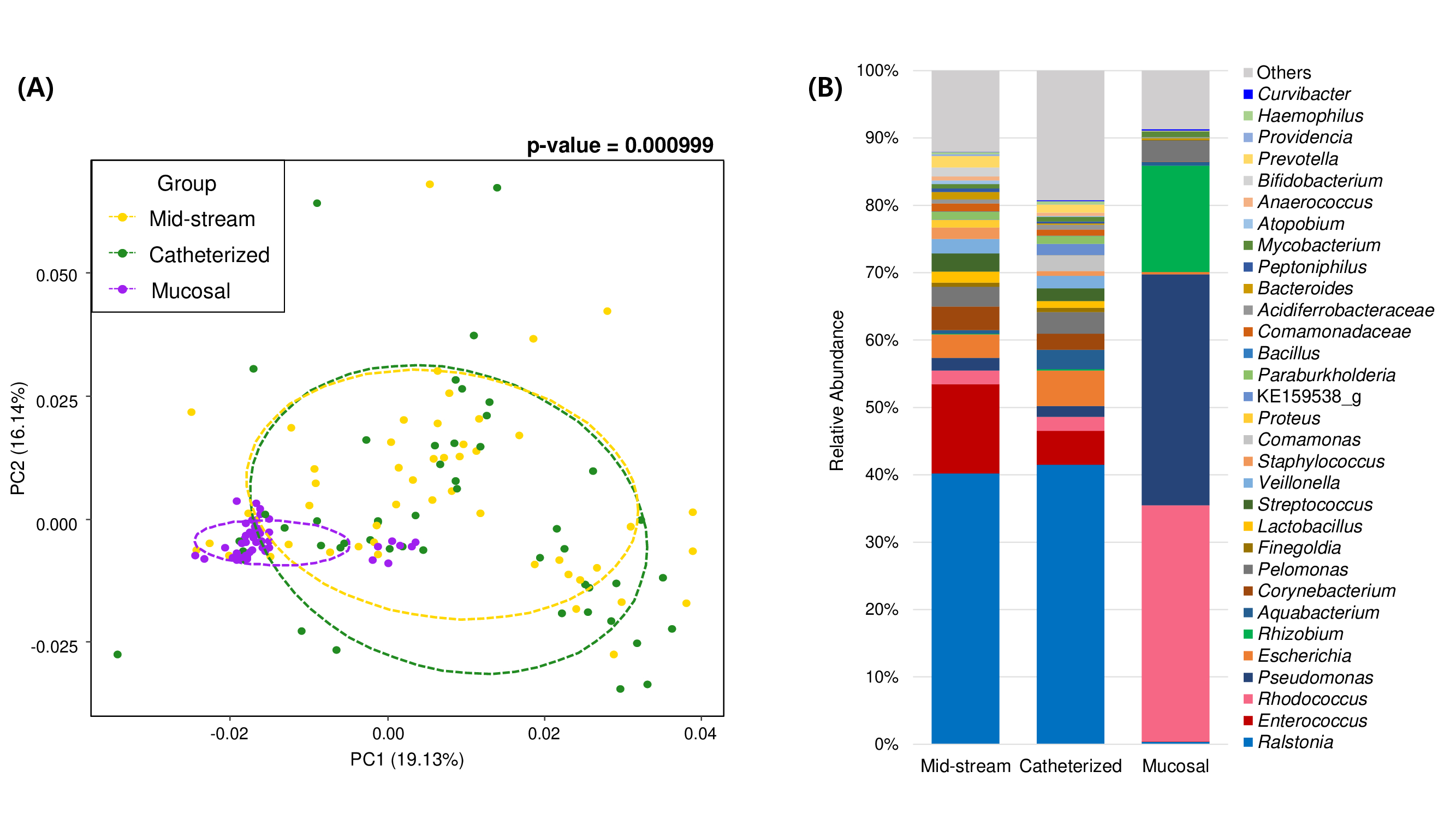

Supplement: Supplementary file 4 — Supplementary Material 4. [file 12866_2025_4367_MOESM4_ESM.jpg]

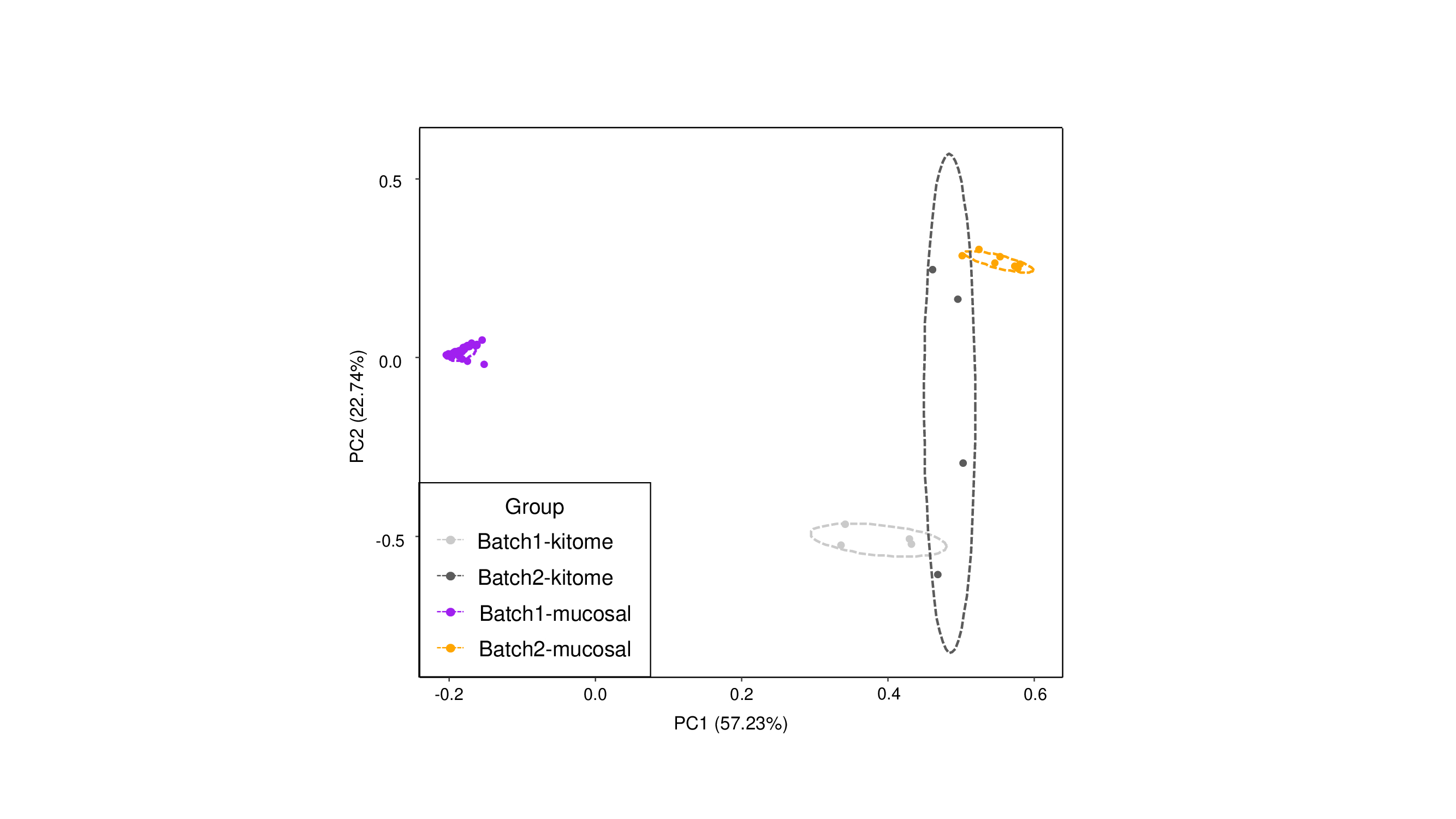

Supplement: Supplementary file 5 — Supplementary Material 5. [file 12866_2025_4367_MOESM5_ESM.jpg]
